# An integrated multi-omics analysis of identifies distinct molecular characteristics in pulmonary infections of *Pseudomonas aeruginosa*

**DOI:** 10.1371/journal.ppat.1011570

**Published:** 2023-08-29

**Authors:** Yang Yang, Teng Ma, Jun Zhang, Yu Tang, Miao Tang, Chaoyu Zou, Yige Zhang, Mingbo Wu, Xueli Hu, Huan Liu, Qianhua Zhang, Yilin Liu, Hongliang Li, Jing Shirley Li, Zhuochong Liu, Jing Li, Taiwen Li, Xikun Zhou

**Affiliations:** 1 Department of Biotherapy, Cancer Center and State Key Laboratory of Biotherapy, West China Hospital, Sichuan University, Chengdu, China; 2 State Key Laboratory of Oral Diseases, National Clinical Research Center for Oral Diseases, Chinese Academy of Medical Sciences Research Unit of Oral Carcinogenesis and Management, West China Hospital of Stomatology, Sichuan University, Chengdu, China; University of Massachusetts Chan Medical School, UNITED STATES

## Abstract

*Pseudomonas aeruginosa* (*P*. *aeruginosa*) can cause severe acute infections, including pneumonia and sepsis, and cause chronic infections, commonly in patients with structural respiratory diseases. However, the molecular and pathophysiological mechanisms of *P*. *aeruginosa* respiratory infection are largely unknown. Here, we performed assays for transposase-accessible chromatin using sequencing (ATAC-seq), transcriptomics, and quantitative mass spectrometry-based proteomics and ubiquitin-proteomics in *P*. *aeruginosa*-infected lung tissues for multi-omics analysis, while ATAC-seq and transcriptomics were also examined in *P*. *aeruginosa*-infected mouse macrophages. To identify the pivotal factors that are involved in host immune defense, we integrated chromatin accessibility and gene expression to investigate molecular changes in *P*. *aeruginosa*-infected lung tissues combined with proteomics and ubiquitin-proteomics. Our multi-omics investigation discovered a significant concordance for innate immunological and inflammatory responses following *P*. *aeruginosa* infection between hosts and alveolar macrophages. Furthermore, we discovered that multi-omics changes in pioneer factors Stat1 and Stat3 play a crucial role in the immunological regulation of *P*. *aeruginosa* infection and that their downstream molecules (e.g., Fas) may be implicated in both immunosuppressive and inflammation-promoting processes. Taken together, these findings indicate that transcription factors and their downstream signaling molecules play a critical role in the mobilization and rebalancing of the host immune response against *P*. *aeruginosa* infection and may serve as potential targets for bacterial infections and inflammatory diseases, providing insights and resources for omics analyses.

## Author summary

*Pseudomonas aeruginosa* (*P*. *aeruginosa*) is a gram-negative aerobic pathogenic bacterium that is widespread in the environment, can undergo anaerobic metabolic activity under certain conditions, is particularly pathogenic and is often associated with lung infections and high mortality rates. In recent years, molecular studies of the mechanisms of the host immune response to bacterial infection have been well interpreted, but the multi-omics changes of multiple molecules in *P*. *aeruginosa* systematic analysis of the relationship between them has never been uncovered in detail.

In this study, we integrated bioinformatics analysis of ATAC-seq, RNA-seq, proteomics and ubiquitinated proteomics to reveal the landscape of molecular and functional changes of *P*. *aeruginosa* infected lung tissues in multiple dimensions. Moreover, we reveal a host defense mechanism by which the crucially important transcription factors Stat1 and Stat3 may co-operatively regulate the expression of the downstream inflammatory genes.

## Introduction

*Pseudomonas aeruginosa (P*. *aeruginosa)* is a gram-negative pathogenic aerobic bacterium that can metabolize anaerobically under certain conditions and has a high degree of virulence modification and immune bypassing ability. *P*. *aeruginosa* can not only effectively counteract immune mechanisms but may also possess a variety of intrinsic and possibly acquired antibiotic resistance traits [1,2]. With widespread environmental occurrence and strong medical relevance, *P*. *aeruginosa* is a common cause of hospital respiratory infections and morbidity and is particularly pathogenic, often associated with pulmonary infections and high mortality [1,3,4]. In response to invading infections, including *P*. *aeruginosa* infection, the host has evolved a variety of immune defense mechanisms, such as innate and adaptive immune response mechanisms, to recognize and clear pathogens [5]. Alveolar macrophages are representative of the innate immune response in the lung and are the first line of defense against contaminants and harmful bacteria. The high plasticity of alveolar macrophages allows them to play an important role in enforcing the defense of homeostasis in the face of the perturbations of pathogenic bacterial pulmonary infection [6]. Although Stat3 activation during *P*. *aeruginosa* lung infection has been reported to be essential for enhancing the Th17 response and sustaining neutrophil airway inflammation [7,8], systematic analysis of multi-omics changes in multiple molecules, intermolecular interactions, and the interrelationship of target molecules with innate immune cells has never been reported in detail. In recent years, significant improvement has been achieved in immune cells’ ability to spot harmful molecular patterns. However, the functional and regulatory mechanisms underlying ingenious immune mobilization and the balance of immune cells remain to be further studied.

In recent advances, omics methods have provided good opportunities for comprehensive characterization of the dynamic properties of tissues and cells under various pathophysiological conditions. The efficient transposase analysis and chromatin sequencing method (Assay for Transposase-Accessible Chromatin using sequencing, ATAC-seq) has successfully mapped genome-wide open chromatin patterns in a variety of diseases [9], and the transcriptional state in tissue or cell type is often measured by RNA-seq to map the regulatory context of gene expression [10]. Proteomics is an important component of biological research, complementing the information obtained from genomics and transcriptomics, and promises to be a rich source of new targets and activation pathways for new therapies [11]. However, there is no report on the immune activation status and mechanism of *P*. *aeruginosa* pulmonary infection from the perspective of multiple omics.

In this study, we used a multi-omics strategy by integrating ATAC-seq, RNA-seq, proteomics, and ubiquitin-proteomics data to explore important signaling pathways and molecular statuses in mouse lung tissues and alveolar macrophages. To better understand the regulatory mechanisms following host infection with *P*. *aeruginosa*, we explored systematic alterations in chromatin accessibility and gene expression by combining the assay for transposase-accessible chromatin using sequencing (ATAC-seq) with RNA sequencing. Comprehensive analyses were then performed to identify potential regulators and their targets associated with differentially accessible regions (DARs) in mouse lung tissue and mouse alveolar macrophages. Further exploration of the omics changes in critical transcription factors in RNA-seq, proteomics, and ubiquitin-proteomics revealed that ubiquitinated modifications of immune signaling-related proteins, such as Stat1 and Stat3, have a pivotal regulatory role in host innate immune regulation. This integrated analysis can provide us with a valuable resource to identify the variables that best describe the multigroup variation of *P*. *aeruginosa* and other bacterial infections.

## Materials and methods

### Ethics statement

Eight-week-old female C57BL/6 mice were purchased from Beijing Huafukang Bioscience Co. Ltd. (Beijing, China) and housed in a specific-pathogen-free facility at the State Key Laboratory of Biotherapy, Sichuan University. All animal experiments were reviewed and approved by the Ethics Committee of the State Key Laboratory of Biotherapy, West China Hospital, Sichuan University (No. 20140104) and carried out in compliance with institutional guidelines concerning animal use and care at Sichuan University.

### Bacterial strains and macrophage cell lines

Wild-type *Pseudomonas aeruginosa* PAO1, obtained from Dr. S. Lory (Harvard Medical School), was routinely cultured in LB (Luria-Bertani) broth with shaking (220 r.p.m.) at 37°C. The mouse alveolar macrophage MH-S cell line (ATCC CRL-2019) was purchased from the American Type Culture Collection (ATCC) and maintained in RPMI-1640 and DMEM (Gibco) supplemented with 10% FBS (Gibco).

### Mouse infection model

In the mouse model, overnight-cultured *P*. *aeruginosa* was collected and diluted in sterile saline to an optical density (600 nm) of 0.5. Ketamine (50 ml^-1^) in sterile saline was injected intraperitoneally to anesthetize C57BL/6 female mice. A total of 1.0 × 10^7^ CFU planktonic bacterial cells were washed three times, suspended in 50 μl of sterile saline, and then injected into the trachea of each mouse [12]. For control injection in mice, *P*. *aeruginosa* was replaced with PBS to complete the same operation. The mice were killed 12 hours after treatment, and to balance the heterogeneity of the samples, we used lung tissue from three mice as one biological replicate, with two biological replicates from the control (TC) and infected (TP) groups subjected to ATAC-seq and proteomic sequencing and three biological replicates subjected to RNA-seq and ubiquitin proteomic sequencing.

### Cell infection model

The widely used alveolar macrophage line, MH-S, was infected with *P*. *aeruginosa* (MOI = 50) for half an hour and washed three times with PBS. Then, the cells were subsequently treated with 100 μg/ml gentamicin for another one and a half hours. The control cells were prepared with the same procedure except *P*. *aeruginosa* was replaced with PBS. Then, the cells were collected for RNA-seq and ATAC-seq analysis. For cell samples, two samples from the control group (CC) and the infection group (CP) were collected for ATAC-seq, and three samples from each group (CC and CP) were prepared for RNA-seq.

### ATAC-Seq library preparation and sequencing

The ATAC-seq library was prepared as described previously, with slight modifications [9]. Briefly, a total of 1 × 10^5^ cells were rinsed with PBS. The cell pellets were resuspended in 50 μL of lysis buffer, and the nuclei were pelletized by centrifugation at 500 × g for 10 min at 4°C. The supernatant was discarded, and the nuclei were resuspended in 50 μL of reaction buffer containing 2.5 μL of Tn5 transposase and 22.5 μL of TD (Nextera Illumina). Incubate at 37°C for 30 minutes. Labeled DNA was isolated using the MinElute PCR Purification Kit (Qiagen). Ten cycles of library amplification and 2× SPRI Cleanup (Agencourt) were used for purification. Using the LabChip GXII Touch HT (PerkinElmer), library sizes were calculated. NextSeq500 (Illumina) was then used to perform 2 × 75 paired-end sequencing, which produced an average of 50 M reads/sample.

### ATAC-Seq data processing

The quality of sequencing reads for ATAC-seq was evaluated using FastQC (v0.11.9). Unmapped reads were processed with trim galore (v0.6.7) using the parameters "-q 30—phred33—stringency 3—length 35" before attempting to map again using the above parameters. Sequencing reads were mapped to the mouse genome (mm10) by bowtie (v1.3.1) [13] with the parameter "-m1—best—strata -X 2000". Only uniquely mapped paired-end reads with fragment lengths less than 2,000 bp and mapping quality greater than 30 were retained for further analysis. The bam files were then merged and processed using the sambamba (v0.8.1) [14] markdup function to remove duplicate reads and then filter out reads that mapped to the mitochondrial genome and to select unique, non-chrM reads from the bam files using samtools (v1.9) [15]. These subnucleosomal fragments were used to call peak summits for each replicate with MACS2 [16] using the parameters "-shift -100 -extsize 200 -nomodel -q 0.01—call-summits—keep-dup all -B". Finally, DiffBind (v3.4.11) [17] was used to assess changes in chromatin accessibility, and the "dba" merge function found all overlapping peaks and produced consistent peaks, allowing "dba.count" to normalize the consistent peak dataset for pairwise comparisons using the DESeq2 algorithm with a false discovery rate (FDR)-adjusted *p* value less than 0.05 to filter out differentially accessible regions (DARs). To normalize and visualize the data, the BAM files were converted to bigWig format using RPKM’s "bamCoverage" [18]. To annotate the positions of ATAC-Seq peaks based on important genomic features, we used the annotatePeaks.pl script in HOMER (v4.11) [19] to assign their BED files to promoter-TSS (defined by default as -1 kb to +100 bp of the transcription start site), TTS (defined by default as -100 bp to +1 kb of the transcription stop site), introns, intergenic regions, exons, etc. The "dba.plotProfile" function in DiffBind was used to generate heatmaps and average profiles, as well as average profiles extending to ± 1.5 kb region around the center of the DARs. The number of genomic originals annotated from mouse lung tissues and MH-S cell DARs were plotted as histograms using ggplot2 [20].

### RNA-Seq library preparation and sequencing

For RNA extraction, a 1% agarose gel was used to detect RNA degradation and contamination. RNA purity was checked using a NanoPhotometer spectrophotometer (IMPLEN), and RNA integrity was subsequently assessed using the Bioanalyzer 2100 System (Agilent) RNA Nano 6000 Assay Kit. Sequencing libraries were generated using the NEBNext Ultra RNA Library Prep Kit for Illumina (NEB, E7530L), and index codes were added to the attribute sequences of each sample and sequenced using the TruSeq PE Cluster Kit v3-cBot-HS (Illumina, PE-401-3001) on the cBot Cluster Generation System to cluster the index-coded samples. After cluster generation, library preparations were sequenced on the Illumina NovaSeq platform to generate 150 bp paired-end reads.

### RNA-Seq data processing

RNA-Seq reads were trimmed using Trim Galore to remove sequencing adapters (v0.6.7), and FastQC (v0.11.9) software was used to assess reads quality. Reads were mapped to the mouse reference genome (mm10) using HISAT2 software (v2.2.1) [21]. The mapped reads were assembled into transcripts or genes by featureCounts (v2.0.2) [22] software with genome annotation based on the mm10.gtf file. Features with zero counts across all samples were eliminated. Raw counts were normalized with differential expression analysis by DESeq2 [23]. Transcripts per kilobase of exon model per million mapped reads (TPM) were also calculated. In lung tissues, differentially expressed transcripts were identified when the FDR-adjusted *p* value was less than 0.01, the fold change (FC) was at least 2 and the group mean TPM score was higher than 10. In MH-S cells, transcripts were retained when the adjusted *p* value was less than 0.01 and FC was at least 1, and group means TPM scores higher than 1 transcript were retained. The R package ggplot2 was used to visualize the expression values of genes in lung tissues and MH-S cells.

### Protein extraction and tryptic digestion

The samples were separately ground into cell powder with liquid nitrogen before being transferred to a 5-mL centrifuge tube. Then, four volumes of lysis buffer (8 M urea, 1% protease inhibitor cocktail, 50 μM PR-619) were added to the cell powder, followed by sonication for three minutes on ice using a high-intensity ultrasonic processor (Scientz) (Note: For PTM experiments, inhibitors were also added to the lysis buffer, 50 μM PR-619 for ubiquitination). The remaining debris was removed by centrifugation at 12,000 × g for 10 minutes at 4°C. Finally, the supernatant was collected, and the protein content was measured using a BCA kit as directed by the manufacturer.

### Proteomics sample preparation

Trypsinized peptides were desalted with Strata X C18 (Phenomenex) and then vacuum freeze-dried. Peptides were solubilized with 0.5 M TEAB. Peptides were solubilized and labeled according to the TMT kit operating instructions. Tryptic peptides were fractionated by high pH reversed-phase HPLC using an Agilent 300Extend C18 column (5 μm particles, 4.6 mm i.d., 250 mm length). Briefly, the peptides were first separated into 60 fractions using a gradient of 8% to 32% acetonitrile (pH 9.0) over 60 min. The peptides were then combined into 18 fractions, which were vacuum freeze-dried for subsequent operations.

### Proteomics LC-MS/MS data acquisition

The peptides were separated by an ultra-performance liquid chromatography system and then injected into an NSI ion source for ionization and analysis by Orbitrap Fusion mass spectrometry. The ion source voltage was set to 2.0 kV, and the peptide parent ions and their secondary fragments were detected and analyzed using a high-resolution Orbitrap. The primary mass spectrometry scan range was set to 350–1,550 m/z, and the scan resolution was set to 60,000, while the secondary mass spectrometry scan range was fixed at 100 m/z, and the secondary scan resolution was set to 15,000. The dynamic exclusion time of the tandem mass spectrometry scan was set to 200 ms. The dynamic exclusion time of the tandem mass spectrometry scan was set to 30 s to avoid duplicate scans of the parent ion. M2 spectrometry data were searched with MaxQuant (v1.5.2.8) [24]. The search parameter settings were as follows: the database was SwissProt [25] Mouse, a reverse library was added to calculate the false discovery rate (FDR) due to random matching, and a common contamination library was added to the database to eliminate the effect of contaminating proteins in the identification results; the digestion method was set to trypsin/P. The mass tolerance for precursor ions was set as 20 ppm in the first search and 5 ppm in the main search, and the mass tolerance for fragment ions was set as 0.02 Da. The quantification method was set to TMT-6plex, and the FDR for protein identification and PSM identification was set to 1%.

### Proteomics data analysis

Protein abundance was obtained after quantitative analysis using MaxQuant to remove anti-library and contaminant proteins. After removing proteins with missing features (zero counts for all samples), the data were logarithmic and analyzed for differential expression using Limma [26]. Differentially expressed proteins (DEPs) were defined by a *p* value < 0.05 with FC > 1. Relative protein abundance of lung tissues plotted as heatmaps and bar graphs using ggplot2 in R.

### Sample preparation for ubiquitin-proteomics

To precipitate protein, the sample was slowly added to a final concentration of 20% (m/v) TCA, vortexed to mix, and incubated for 2 hours at 4°C. Centrifugation at 4500 × g for 5 minutes at 4°C was used to collect the precipitate. The precipitated protein was washed three times with precooled acetone and dried for one minute. The protein sample was then ultrasonically dispersed after being redissolved in 200 mM TEAB. For the initial digestion, trypsin was added at a 1:50 trypsin-to-protein mass ratio overnight. The sample was reduced for 60 minutes at 37°C with 5 mM dithiothreitol and alkylated for 45 minutes at room temperature in darkness with 11 mM iodoacetamide. The peptides were then desalted using a Strata X SPE column.

### Ubiquitin-proteomics DIA data acquisition

To enrich modified peptides, tryptic peptides dissolved in NETN buffer (100 mM NaCl, 1 mM EDTA, 50 mM Tris-HCl, 0.5% NP-40, pH 8.0) were incubated at 4°C overnight with gentle shaking with prewashed antibody beads (PTM1104, PTM Bio). The beads were then washed four times in NETN buffer and twice in H2O. The bound peptides were eluted from the beads with 0.1% trifluoroacetic acid. Finally, the eluted fractions were combined and vacuum-dried. The resultant peptides were desalted using C18 ZipTips (Millipore) according to the manufacturer’s instructions for LC-MS/MS analysis. The peptides were dissolved in 0.1% FA and separated by a NanoElute UHPLC system (Bruker Daltonics). TimsTOF Pro mass spectrometry was used to separate the peptides after they were separated using a UHPLC system.

### Ubiquitin proteomics data analysis

Ubiquitinated site protein abundance was obtained after quantitative analysis using Spectronaut (v 17). After removing proteins with missing features (zero counts for all samples), the data were logarithmically processed and analyzed for differential expression using Limma. Proteins with differentially expressed ubiquitinated sites (DUPs) were screened and retained when the FDR-adjusted *p* value was less than 0.1 and the FC was at least 1. Relative ubiquitinated site protein abundance of lung tissues plotted as heatmaps and bar graphs using ggplot2 in R.

### Correlation analysis of DARs and DEGs

DARs were annotated with genomic features using the "annotatePeaks.pl" function in HOMER. DARs of neighboring genes (< 10 kb distance from the TSS) were then overlaid with the DEGs identified by RNA-seq. Venn plots, scatter plots, and heatmaps were produced using the Venn Diagram [27] and ggplot2 R packages.

### Correlation of lung tissues with changes in the characteristics of MH-S cells

In chromatin-accessible regions, a comparison of the proportion of genomic elements annotated and reads quantified by RPKM for DARs of lung tissues and MH-S cells. In the transcriptomics, the DEGs correlation between lung tissues and MH-S cells was also compared. Genes with common differences between tissues and cells were identified using the iUUCD database [28] to identify the component of the ubiquitin-proteasome system to which they belong. Scatter plots and pie charts, as well as Pearson correlation coefficients and *p* values, were generated using the ggplot2 R package.

### Enrichment analysis

Enrichment analysis of Gene Ontology (GO), Reactome Database, and KEGG pathways were implemented by the clusterProfiler [29] and ReactomePA [30] R packages. An FDR-adjusted *p* value < 0.05 for enrichment terms and pathways was considered significant, and the enrichment of top GO terms and KEGG pathways were the key features associated with *P*. *aeruginosa* infection elicitation.

### Characteristics of the TFs with altered multi-omics profiles

For motif enrichment analysis of DARs, we used the "findMotifsGenome" function of HOMER to discover known motifs surrounding DARs and to identify binding sites for a certain TF with the identification parameter "-size 200 -mask". The motifs were retained only when the *p* value was less than 0.05. To explore the interactions between mouse alveolar macrophages and tissues after *P*. *aeruginosa* infection, differentially expressed transcription factors (DETFs) between tissues and cell lines were retained, and DETFs in at least three omics were screened for subsequent exploration. Multiple signature-altering transcription factors were identified based on multi-omics data, and neighboring genes were screened using a 10 kb window on either side of the peak.

### Statistical analysis and data visualization

If not specified, R language was used to calculate statistics and generate plots in this study. The fold change, *p* value, and adjusted *p* value were calculated in the analysis. Genomic signal traces were extracted and visualized using the Integrative Genomics Viewer (IGV) [31].

## Results

### Overview of the integrated multiple omics in the lung tissues of mice infected with *P*. *aeruginosa*

**Fig 1:**
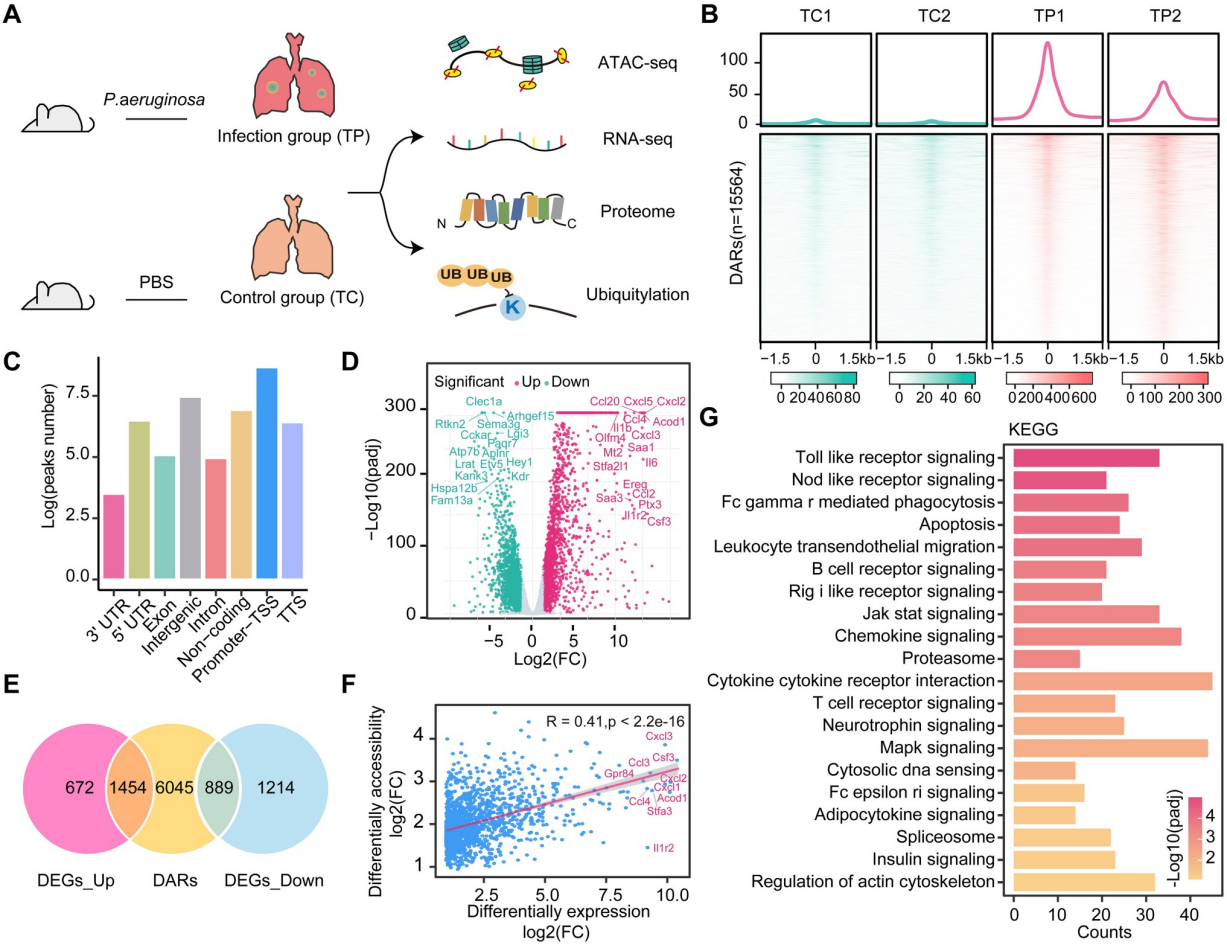
Analysis of differentially expressed genes (DEGs) and differentially accessible regions (DARs) in mouse lung tissues. (A) Overview of the study design. Four omics indicators with *P*. *aeruginosa*-infected lung tissue were integrated to dissect chromatin accessibility, transcripts, protein, and ubiquitinated protein abundance and alteration. (B) The average read density (top) and heatmap (bottom) representation of normalized ATAC-seq signals over DARs in the infection and control groups. Heatmaps display a read signal across 15,567 accessible regions. Signals within 1.5 kb of the center of DARs are shown in descending order. (C) Bar chart showing annotation of DARs to genomic features: 3′ UTR, 5′ UTR, exon, intergenic regions, introns, non-coding regions, promoter-TSS, and TTS. Promoter-TSS regions are defined as peak summits located up to 1 kb upstream and 100 bp downstream of the TSS. (D) Volcano plot showing the transcript levels of DEGs between the infection and control groups. Significantly upregulated 2126 genes are shown as red dots, whereas 2103 downregulated genes are shown as blue dots. Filtered by adjusted *p* value < 0.01, the top significantly up- and downregulated genes are highlighted in the text according to log2(FC). (E) Venn diagram showing the overlap between nearby genes from DARs and DEGs. (F) Correlation analysis between DARs and their nearest upregulated DEGs. Each blue dot represents a gene that is significantly differentially expressed and associated with chromatin accessibility changes. Based on log2(FC), the top-ranked DEGs are labeled and shown in the text. Pearson’s correlation coefficient (r) and the associated *p* value are displayed. (G), Key KEGG pathways associated with upregulated DEGs related to DARs.

To reveal the changes in open chromatin, gene expression, proteomics, ubiquitin-proteomics, and their relationship with *P*. *aeruginosa* infection, we established an infection model by transtracheal injection of planktonic wild-type *P*. *aeruginosa* PAO1 (reference PAO1 strain) for 12 hours as described in our previous report [12]. Then, we collected lung tissues from healthy control (TC) and *P*. *aeruginosa*-infected (TP) mice for each condition and dissociated these samples for ATAC-seq, RNA-seq, proteomics, and ubiquitin-proteomics sequencing (Fig 1A).

### Key signaling pathway analysis of differentially expressed genes (DEGs) associated with chromatin accessibility in lung tissues infected with *P*. *aeruginosa*

We first used ATAC-seq data to analyze the open chromatin regions. There was reasonable reproducibility between samples, and the chromatin in the samples was also accessible to transposase to the same extent (S1A–S1C Fig). We identified 18023 open chromatin regions and 15564 DARs sites in mouse lung tissues to observe the altered chromatin accessibility corresponding to transcriptional alterations (Figs 1B and S1D, S1 Table). Previous studies have found that open chromatin at transposase hypersensitive sites predicts active transcription [9,32,33], and our findings are consistent with previous studies in a variety of organisms. Our results show that ATAC-seq signaling is significantly enriched in a region ±1 kb around the TSS, implying that most cis-regulatory regions are located near the core promoter of the gene. Moreover, the distribution of DARs on functional genomic elements revealed that promoter-TSS and intergenic regions are also preferentially accessible to Tn5 transposase (Fig 1C), with a relatively large number of promoter-TSS regions exhibiting significantly different accessibility, hinting that most DARs resulting from infection may represent proximal regulatory elements, such as promoters, located close to the genes they control, favoring gene transcription.

RNA-seq was performed to investigate potential genes associated with *P*. *aeruginosa*-infected lung tissues. Furthermore, to identify candidate genes associated with *P*. *aeruginosa* infection, we defined DEGs significantly associated with *P*. *aeruginosa* infection by fold change > 2 and *p* value < 0.01, with a mean TPM > 10 for at least one group. A total of 4229 DEGs were identified, of which 2126 genes were upregulated and 2103 genes were downregulated (Figs 1D, S2A and S2B, S2 Table). We found that some macrophage-associated inflammatory genes were upregulated, such as Cxcl2, Cxcl3, Cxcl5, Acod1, Il1r2, and Il6. Previous studies have shown that Acod1 and Il6 play an important role as immune regulators in maintaining homeostatic balance in the host and that the accumulation resulting from the infection response confers a powerful proinflammatory response to the host [34,35]. Our results may suggest that macrophages play a role in *P*. *aeruginosa* infection by triggering innate and inflammatory immune responses in the host.

The open chromatin regions were more likely to affect the expression of nearby genes, and we found that the majority of DEGs in chr2, chr5, chr7, and chr11 (S2C Fig) located in these regions were significantly downregulated (S2D–S2G Fig), indicating a close connection between changes in chromatin accessibility and concomitant differential gene expression. Therefore, we overlapped the nearest genes of DARs located within 10 kb of the TSS with DEGs and obtained 1454 upregulated genes and 889 downregulated genes associated with changes in chromatin open regions (Fig 1E). Upon further analysis, it was confirmed that changes in upregulated DEGs expression were positively correlated with DARs 10 kb from the TSS (Pearson’s r = 0.41), suggesting that altered chromatin accessibility contributes to the differential expression of genes associated with *P*. *aeruginosa*-infected lung tissues, such as Cxcl3, Il1r2, and Acod1 (Fig 1F). These genes have an important function in immunological regulation and the prevention of microbial infections [36–38]. It is worth adding that the downregulated DEGs were not significantly related to the signal values for the open accessibility of DARs (S2H Fig).

To identify important DEGs and signaling pathways connected to DARs in the lung tissues of a mouse infected with *P*. *aeruginosa*, we conducted an enrichment analysis of DEGs connected to DARs. KEGG enrichment results showed that DARs associated with upregulated DEGs were significantly enriched in Toll-like receptors, Nod-like receptors, Fcγ receptors (FcγRs)-mediated phagocytosis, and JAK/STAT signaling pathways, whereas DARs associated with downregulated DEGs were mainly involved in the Lysosome, Cell cycle, and Endocytosis (Figs 1G and S2I). By exploring the Reactome database, key DEGs associated with DARs were found to be significantly enriched with Cytokine signaling, Interleukin signaling and Toll-like receptor cascades (S2J Fig). Thus, these signals might coordinate the activation of regulation of host innate immunity as previously reports [39,40]. According to these findings, changes in chromatin accessibility affect the gene expression of components of numerous signaling pathways and play a key role in *P*. *aeruginosa*-infected lung tissues.

### Landscape alterations of accessible regions and related DEGs in *P*. *aeruginosa*-infected alveolar macrophages

**Fig 2:**
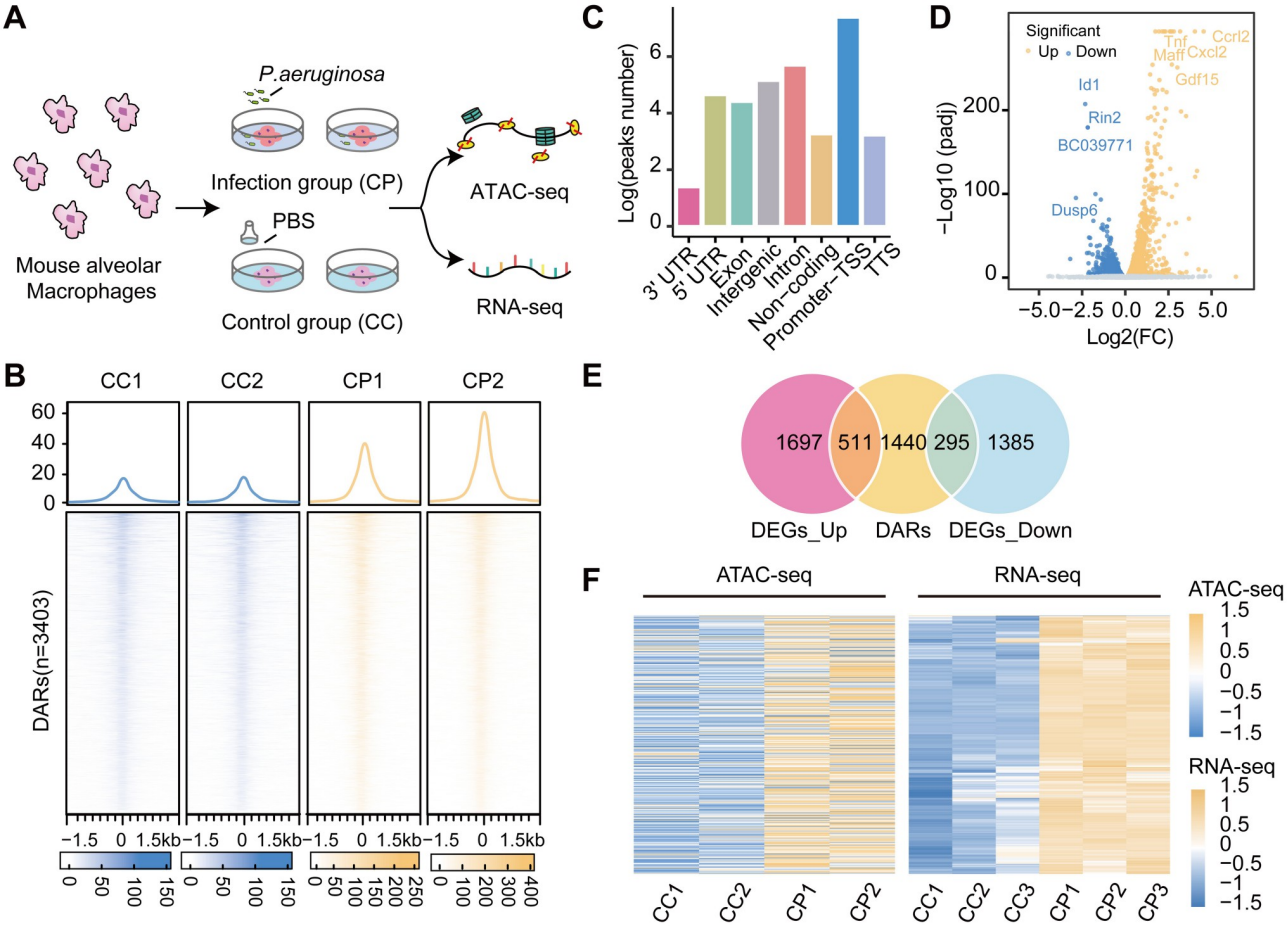
Identification and analysis of DEGs and DARs in *P*. *aeruginosa*-infected mouse alveolar macrophages. (A) Overview of the study design. Two biological replicates infected with *P*. *aeruginosa* were evaluated to profile chromatin accessibility, and three biological replicates infected with *P*. *aeruginosa* were evaluated to profile transcript abundances. (B) The average read density (top) and heatmap (bottom) representation of the normalized ATAC-seq signal over DARs in the infection and control groups. Heatmaps show a read signal of 3403 available locations. Signals within 1.5 kb of the center of the DARs are shown in descending order. (C) Bar chart showing annotation of DARs to genomic features: 3′ UTR, 5′ UTR, exon, intergenic regions, introns, non-coding regions, promoter-TSS, and TTS. Promoter-TSS regions are defined as peak summits located up to 1 kb upstream and 100 bp downstream of the TSS. (D) Volcano plot showing the transcript levels of DEGs between the infection and control groups. A total of 2208 significantly upregulated genes are shown as red dots, whereas 1680 downregulated genes are shown as blue dots. Filtered by adjusted *p* value < 0.01, the top significantly up- and downregulated genes are highlighted in the text according to log2(FC). (E) Venn diagram showing the overlap between nearby genes from DARs and DEGs. (F) Heatmap depicting the expression levels of overlapping genes around upregulated DEGs and DARs.

The alveolar macrophages represent the first line of immune cells in the lung and infiltrated monocytes and macrophages play an important role in the fight against pathogens infection. The abundance of macrophage infiltration in lung tissue was predicted using multiple methods with the data of bulk RNA-seq [41]. The abundance of macrophages was affected by *P*. *aeruginosa* infection and was significantly increased in the infected group (S4A Fig). Therefore, the current study performed ATAC-seq and RNA-seq sequencing to investigate the molecular changes at the cellular level by using *P*. *aeruginosa*-infected alveolar macrophages (Fig 2A). We explored the open chromatin state in *P*. *aeruginosa*-infected alveolar macrophages with a total of 10,930 accessible regions and 3,403 regions of DARs (Fig 2B). Additionally, we found that most of these DARs were in the promoter-transcription start site (promoter-TSS) region and intragenic (Fig 2C and S3 Table). Interestingly, the results for chromatin-open regions in *P*. *aeruginosa*-infected alveolar macrophages were similar to those observed in *P*. *aeruginosa*-infected lung tissues. Furthermore, we set up three biological replicates per group to perform RNA-seq to identify genes that might be associated with *P*. *aeruginosa*-infected alveolar macrophages. Using similar processing to that of lung tissue, we identified a total of 3888 DEGs, of which 2208 were upregulated and 1680 were downregulated (Figs 2D, S3A and S3B, and S4 Table).

Likewise, we further assigned DARs to the nearest genes to explore the potential relationship between DARs and DEGs in alveolar macrophages infected with *P*. *aeruginosa*. Integrating ATAC-seq and RNA-seq data, a total of 511 upregulated and 295 downregulated DEGs were identified with DARs (Fig 2E). Within open chromatin regions, we found that DARs and DEGs tended to have higher expression levels in *P*. *aeruginosa*-infected alveolar macrophages (Fig 2F, and S5 Table). Similarly, infection-related changes in chromatin accessibility were closely associated with the differential expression of upregulated DEGs (S3C and S3D Fig), and this finding was largely consistent with the results in infected mouse lung tissues. In addition, we performed functional and pathway enrichment analyses of DEGs associated with DARs and observed remarkable changes in the expression of important DEGs linked with DARs in *P*. *aeruginosa*-infected alveolar macrophages, which may be crucial for altered immune responses. By exploring the Reactome database, we found that key upregulated DEGs associated with DARs were linked to Cytokine signaling and Interleukin signaling in the immune system (S3E Fig). In KEGG pathway enrichment analysis, we found that upregulated DEGs associated with DARs were responsible for the regulation of the Actin cytoskeleton, the JAK/STAT signaling pathway and the MAPK signaling pathway. Among them, the control of the regulation of the Actin cytoskeleton is associated with the differentiation and productivity of macrophages, and the JAK/STAT and MAPK signaling pathways are classic inflammatory immunological pathways (S3F Fig) [42,43]. In conclusion, our exploration of DEGs in infection-associated chromatin open regions contributes to the understanding of important functions and pathways related to *P*. *aeruginosa* infection and immunity, which provides strong evidence for understanding the molecular mechanisms of bacterial infections.

### Integration of genomic and transcriptomic data from mouse lung tissues and alveolar macrophages reveals functional and molecular alterations associated with ubiquitination

**Fig 3:**
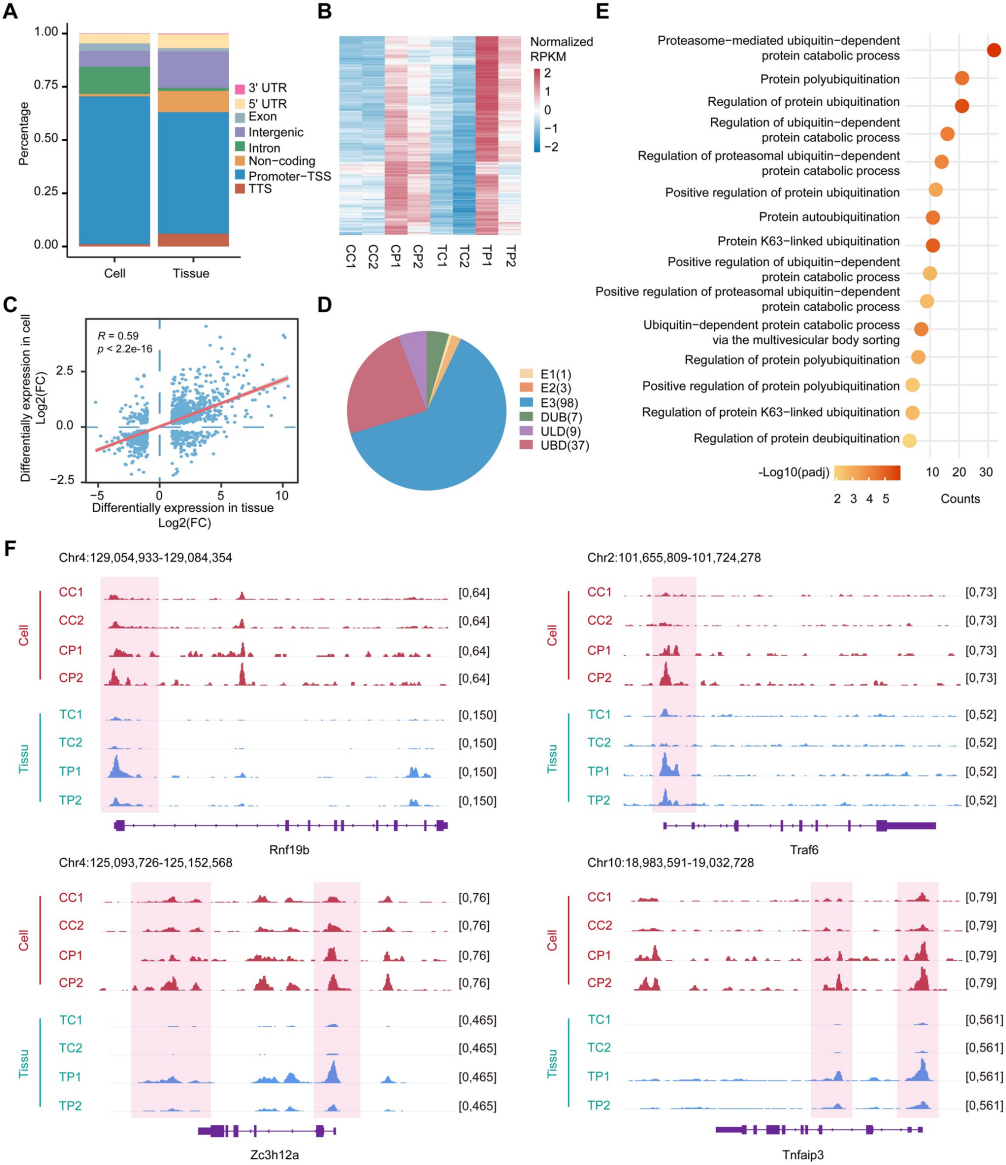
Molecular interactions between lung tissues and alveolar macrophages. (A), Proportions of DARs within genomic regions in lung tissues and alveolar macrophages: 3′ UTR, 5′ UTR, exon, intergenic regions, introns, non-coding regions, promoter-TSS, and TTS. (B) Heatmap representation of normalized ATAC-seq signals of DARs shared by lung tissues and alveolar macrophages. (C) Correlation analysis of DEGs between lung tissues and mouse alveolar macrophage groups. Each blue dot represents a gene that is significantly differentially expressed and correlates with changes in chromatin accessibility. Pearson’s correlation coefficient (r) and associated *p* values are shown. (D) Pie chart showing the components of the ubiquitin-proteasome system of DEGs shared by mouse lung tissues and alveolar macrophages. (E) Bubble plot illustrating the significant enrichment of the ubiquitination function in the DEGs shared by mouse lung tissues and alveolar macrophages. The position corresponds to the number of genes, whereas color denotes significance. (F) IGV Browser view of four upregulated accessible chromatin regions of the components of the ubiquitin-proteasome system in alveolar macrophages and lung tissues.

In our study of DEGs in chromatin-open regions, we identified significant similarities in the molecular and functional characteristics of mouse lung tissues *in vivo* and alveolar macrophage cell lines *in vitro*, and we then further explored the molecular interactions between *P*. *aeruginosa*-infected alveolar macrophages and lung tissues. We first summarized the distribution of genomic elements of detected DARs in lung tissues and alveolar macrophages following *P*. *aeruginosa* infection (Fig 3A). As in mouse lung tissues, a considerable part of the promoter-TSS region was found in alveolar macrophages. Meanwhile, by observing the changes in chromatin open signal intensity in both *P*. *aeruginosa*-infected mouse lung tissues and alveolar macrophages, we found that the open signal was significantly higher in the *P*. *aeruginosa*-infected group than in the control group (Fig 3B, and S6 Table). Then, we analyzed the correlation of fold changes in DEGs and further demonstrated the consistency of differential changes in DEGs in lung tissues and alveolar macrophages infected with *P*. *aeruginosa* (Fig 3C). We also found that in *P*. *aeruginosa*-infected mouse lung tissues and alveolar macrophages, coexisting DEGs were significantly enriched for ubiquitination-related functions and components belonging to the ubiquitin-proteasome system, with ubiquitin ligase (E3) being particularly prominent (Fig 3D and 3E). In particular, proteasome-mediated ubiquitin-dependent proteolytic processes, protein polyubiquitination, and regulation of protein ubiquitination pathways play a key role in the maintenance of protein homeostasis following *P*. *aeruginosa* infection. Moreover, we discovered these molecules that not only increase signaling in the open region following *P*. *aeruginosa* infection but are also involved in the regulation of a variety of important ubiquitination functions. Of these, Rnf12b and Traf6 are ubiquitinated ligases (E3), while Tnfaip3 (also known as A20) and Zc3h12a are deubiquitinated enzymes (DUBs), and the differential expression patterns of these molecules correlate with altered chromatin accessibility (Figs 3F, S4B). Notably, Rnf12b has been identified as a key regulator of signal transducer and activator of transcription 1 (Stat1), as well as a component of the innate immune response [44].

### Characterization of the proteomics and ubiquitin-proteomics in lung tissues infected with *P*. *aeruginosa*

**Fig 4:**
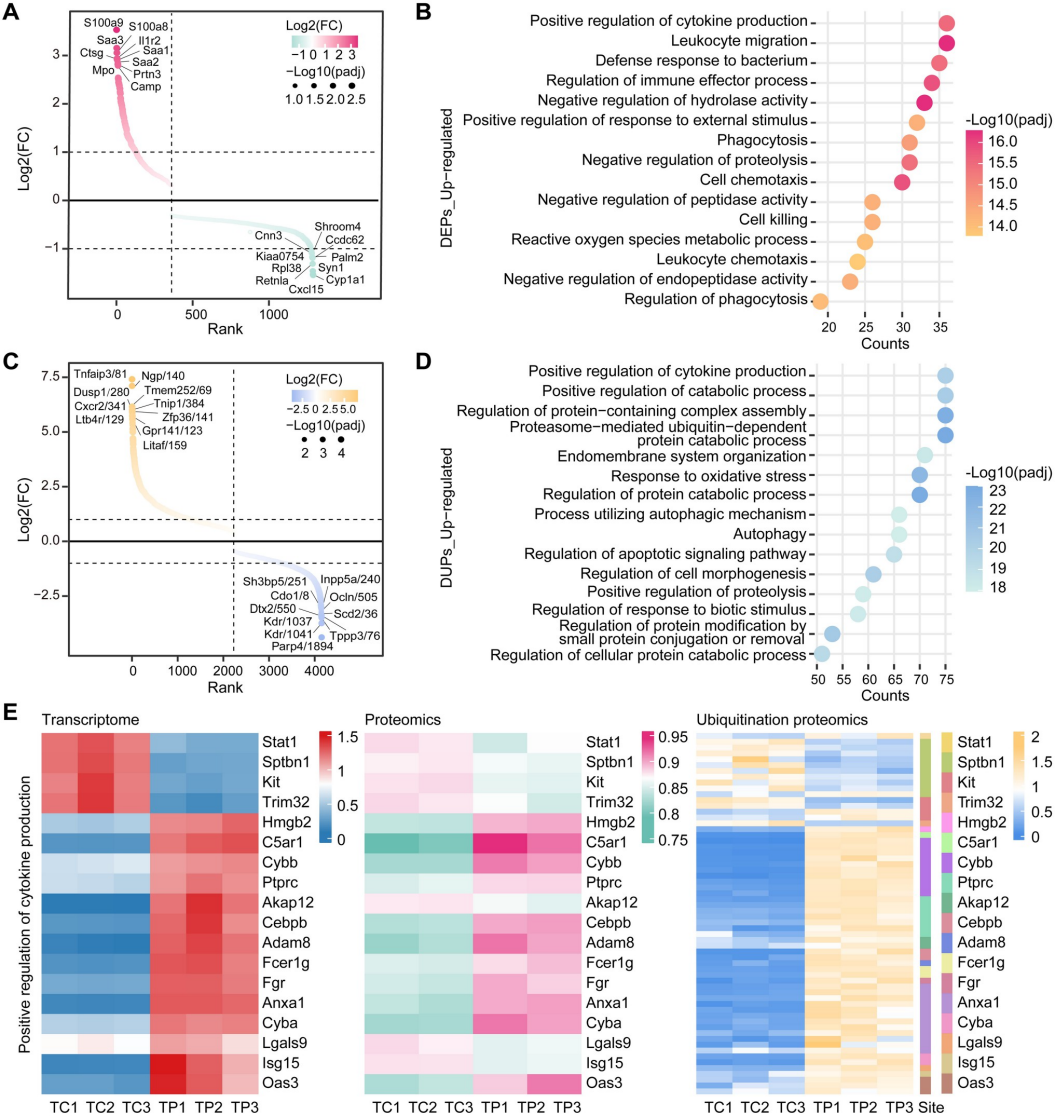
Identification and analysis of DEPs and DUPs in *P*. *aeruginosa*-infected lung tissues. (A) The waterfall plot of the DEPs arrangement is sorted by log2(FC), with upregulated expression shown in red and downregulated expression shown in green. (B) The GO function enrichment analysis for upregulated DEPs is represented in a bubble plot. Color represents significance, and position represents the number of enriched genes. (C) The waterfall plot of the DUPs arrangement is sorted by log2(FC), with upregulated expression shown in yellow and downregulated expression shown in blue. (D) Results of the study of GO functions that are enriched for upregulated DUPs are displayed in a bubble plot. Position indicates the number of enriched genes, whereas color denotes significance. (E) Heatmap showing the abundance of molecules with altered transcriptomics, proteomics, and ubiquitin proteomics in the positive regulation of cytokine production.

Based on combined genomic and transcriptomic analyses that identified genetic and functional alterations associated with ubiquitinated modifications, we performed additional proteomic and ubiquitin-proteomic sequencing for mouse lung tissues. We identified and characterized 1276 differentially expressed proteins (DEPs) and 4142 differentially expressed ubiquitinated sites (DUPs) and performed enrichment analysis on DEPs and DUPs (Figs 4A–4D, S4C and S4D, S7 and S8 Table). In addition, key proteins and ubiquitinated proteins associated with *P*. *aeruginosa* infection were revealed, for example, the significantly upregulated proteins S100A8, SAA3, and IL1R2 and the significantly upregulated ubiquitinated proteins TNFAIP3, TNIP1, and CXCR2. Among them, the differential protein SAA3 and the differential ubiquitinated protein TNFAIP3 not only act as biomarkers of infection and inflammation but are major determinants of inflammatory status and disease progression in many cases [45,46]. With further analysis, we integrated tissue and cellular transcriptomic data and found that Positive regulation of cytokine production was significantly present in GO enrichment (S5A and S5C Fig), and interestingly, the pathway was equally prominent in proteomic and ubiquitin-proteomic enrichment (Fig 4B and 4D). Subsequently, we combined transcriptomic, proteomic, and ubiquitin-proteomic data from the positive regulation of cytokine production gene set for analysis, and we found 18 molecules that differed in multi-omics expression and that these molecules’ expression was mostly consistent in transcriptomics, proteomics, and ubiquitin-proteomics (Fig 4E). Among them, we found that the omic changes in ADAM8, ANXA1, SPTBN1, and STAT1 were highly correlated with inflammatory cell activation and cytokine production [47–51]. At the same time, we found significant expression of macrophage-associated inflammatory factors in both infected lung tissues and cells (S5B and S5D Fig). In conclusion, the GO annotation identified promising molecules and proposed Positive regulation of cytokine production pathways that may be suitable candidate immunological targets in *P*. *aeruginosa* infection.

### Identification of the potential pioneer transcription factors Stat1 and Stat3 during *P*. *aeruginosa* infection

**Fig 5:**
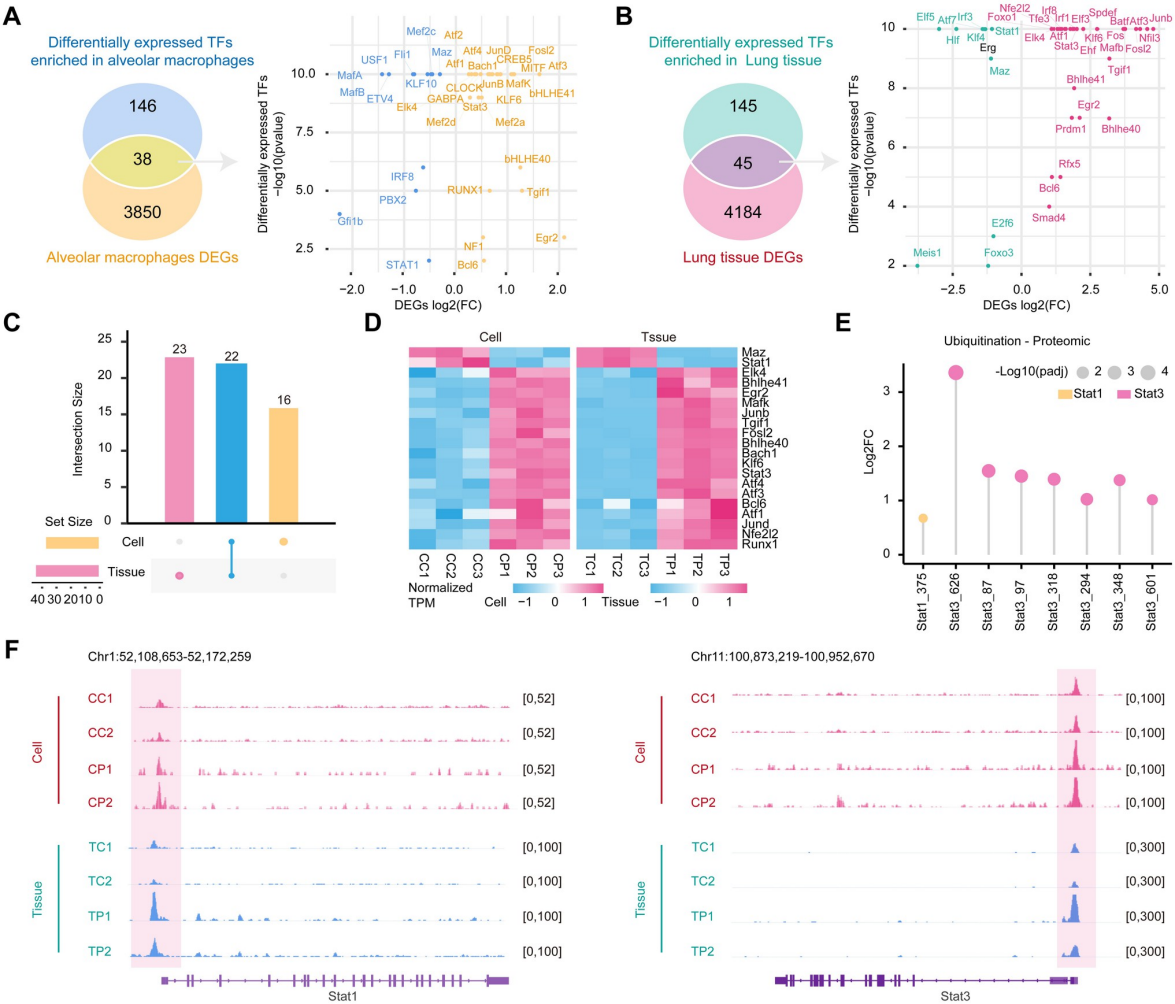
Distinct signatures of TFs in the multiple omics analysis. (A) Dot plot of the transcriptomic differential distribution of differentially expressed TFs (DETFs) in mouse alveolar macrophages. Horizontal coordinates represent log2(FC) of DEGs, yellow is upregulated, blue is downregulated, and vertical coordinates represent motif-enriched TF significance. (B) Dot plot of the transcriptomic differential distribution of differentially expressed TFs (DETFs) in mouse lung tissues. Horizontal coordinates represent the log2(FC) of DEGs; red indicates upregulation, and green indicates downregulation. (C) Multiset visualization depicting the overlap of TFs shared by lung tissues and cells with DEGs from mouse alveolar macrophages and DEGs from lung tissues. (D) Heatmap depicting the gene expression of DETFs shared by lung tissues and alveolar macrophages. The cell is on the left, and the tissue is on the right. (E) Lollipop plots show transcription factors whose abundance in the ubiquitinated proteome is significantly different in both mouse alveolar cells and tissues. (F) IGV browser view shows multiple omics-altered transcription factors in DARs, with mouse alveolar macrophages in the red area and lung tissues in the blue area.

TFs are molecules that regulate gene expression by binding to cis-regulatory specific sequences (called motifs) in target gene promoters or enhancers [52]. In many cases, the chromatin accessibility of DNA is a prerequisite for TF binding, which plays an important role in development and disease [32]. To investigate potential TFs within DARs in *P*. *aeruginosa*-infected mouse lung tissues and alveolar macrophages, we performed TF enrichment analysis using the motif discovery software HOMER. The ± 200 bp flanking sequences around the top of the ATAC-seq peak in DARs were scanned for TF motif occurrence and TF binding sites (TFBS). In the differently accessible regions of the *P*. *aeruginosa*-infected lung tissues and alveolar macrophages, 190 and 184 putative TFs were found by default (p < 0. 05) (S9 and S10 Table). Putative TFs were intersected with the respective DEGs of lung tissue and alveolar macrophages to find DETFs associated with the regulation of chromatin accessibility and gene transcription. A total of 45 DETFs were found in the lung tissues, and 38 DETFs were enrolled in the alveolar macrophages (Fig 5A and 5B). Meanwhile, we identified 22 shared DETFs in lung tissue and alveolar macrophages (Fig 5C). Among them, 20 DETFs with similar expression trends in mouse lung tissues and alveolar macrophages were found, such as Junb, Stat3, Fosl2, and Stat1 (Fig 5D). When the abundance of ubiquitinated proteins of these shared factors was compared, only Stat1 and Stat3 showed significant changes at different levels (Fig 5E). Notably, we found significantly altered chromatin open region signals of Stat1 and Stat3 in infected groups, both in *P*. *aeruginosa*-infected lung tissues and alveolar macrophages (Fig 5F).

Stat1 and Stat3 have been reported to induce the expression of a large number of inflammatory mediators, regulate the biological effects of many other cytokine receptors in various cell types, and act as core transcription factors in various immune responses [53, 54]. Furthermore, Stat1 is an important mediator of M1 macrophage polarization induced by IFN-γ, while IL-10 and IL-6 induce Stat3-mediated expression of genes associated with an M2-like phenotype [55]. Our study revealed similar omics changes in the transcription factors Stat1 and Stat3 in lung tissues and alveolar macrophages in *P*. *aeruginosa*-infected mice, suggesting that Stat1 and Stat3 may play vital roles in host immune defense and immune balance during P. aeruginosa infection.

### The downstream molecules of Stat1 and Stat3 may be important for the balance of the host immune response in *P*. *aeruginosa* infection

**Fig 6:**
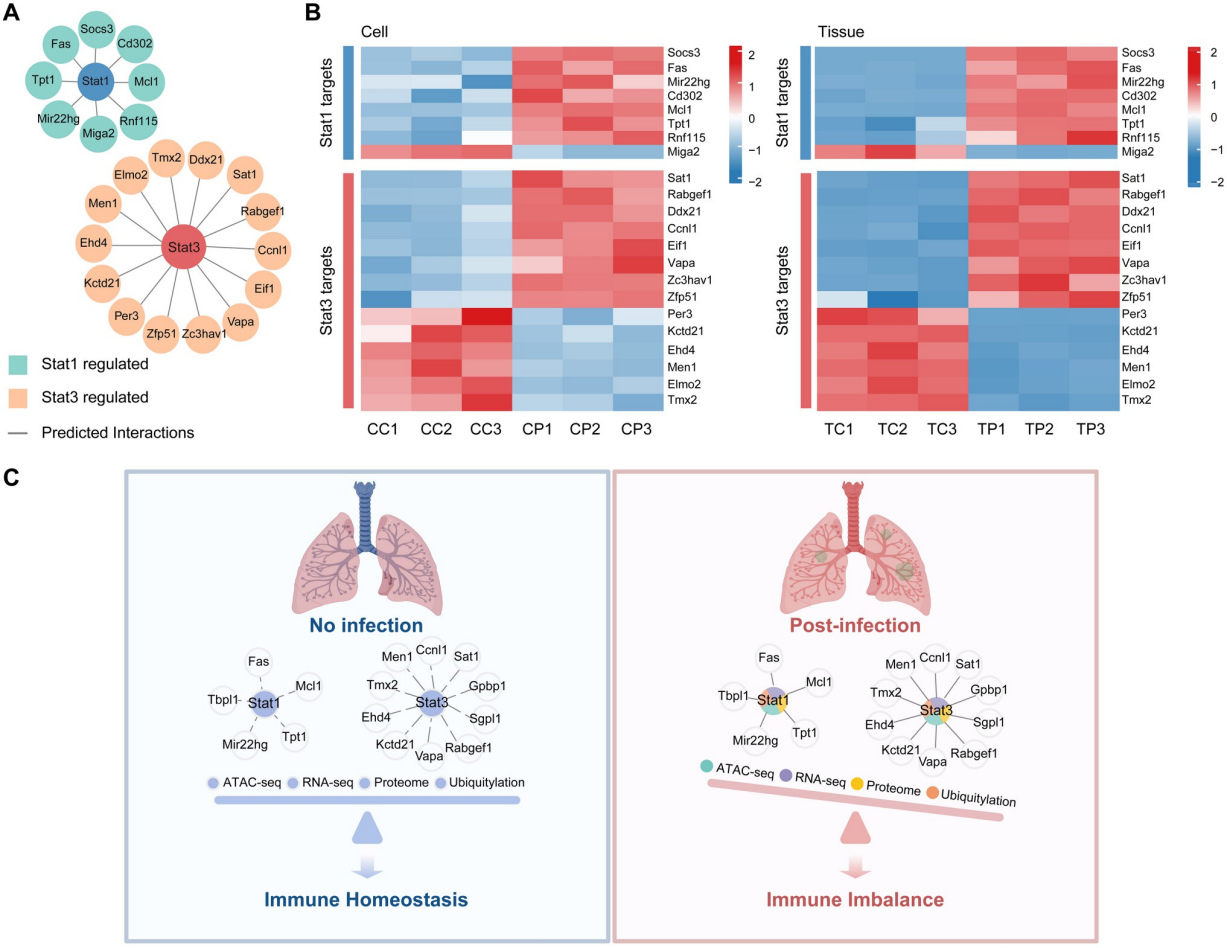
Altered downstream signaling molecules of Stat1 and Stat3 upon *P*. *aeruginosa* infection. (A) Network diagram showing the Stat1 and Stat3 target DEGs in the DARs predicted using Homer. (B) Heatmap showing the gene expression of Stat1 and Stat3 target genes found in mouse alveolar macrophages and lung tissue. (C) A schematic model of Stat1- and Stat3-mediated regulation during pulmonary infection with P. aeruginosa. Lung model created with BioRender.com.

Based on the above molecular analysis, we identified specific alterations in the multi-omics expression of Stat1 and Stat3. Moreover, the altered ubiquitination of Stat1 and Stat3 proteins in proteomics may lead to changes in protein abundance, thereby affecting the transcriptional regulation of downstream molecules. To further characterize the interaction of Stat1 and Stat3 with downstream molecules after *P*. *aeruginosa* infection of the host, we predicted downstream targets based on Stat1 and Stat3 chromatin open regions (Fig 6A), specifically using HOMER to screen the motifs of Stat1 and Stat3 in the promoter region of adjacent DEGs. We identified the coexistence and consistent expression of eight Stat1 downstream genes (Socs3, Fas, Mir22hg, Cd302, Mcl1, Tpt1, Rnf115, and Miga2) and fourteen Stat3 downstream genes (Sat1, Rabgef1, Ddx21, Ccnl1, Eif1, Vapa, Zc3hav1, Zfp51, Per3, Kctd21, Ehd4, Men1, Elom2, and Tmx2) in *P*. *aeruginosa*-infected mouse lung tissues and alveolar macrophages (Fig 6B). Furthermore, several predicted downstream molecules have been reported to be closely associated with inflammation as inflammatory markers, for example, molecules downstream of Stat1, Socs3, Fas and Mcl1; and molecules downstream of Stat3, Sat1, Rabgef1 and Men1 [56–61]. Meanwhile, it has been reported that interactions between Stat1 and Fas, Stat3 and Socs3 are associated with the inflammatory response of the host [62]. These results revealed that low expression of Stat1, high expression of Stat3, and high expression of Fas may together promote the host inflammatory response but this phenomenon was consistently demonstrated in mouse alveolar macrophages and lung tissues.

In summary, a model revealed important roles for Stat1 and Stat3 in innate immune regulation and inflammatory responses (Fig 6C). Multi-omics alterations of Stat1 and Stat3 indicated their central role in the host immune response to *P*. *aeruginosa* infection, and their downstream molecules may be potential targets against bacterial infections and inflammatory diseases.

## Discussion

Although several molecular mechanisms involved in the process of *P*. *aeruginosa* infection have been reported [63,64], inflammation is almost inevitable in bacterial infections, which remains a major unresolved problem in patient management. A growing number of studies have shown that alterations in chromatin accessibility play a key role in disease treatment [32,65] and that chromatin accessibility is a marker for active enhancers, which are cis-regulatory elements of gene expression [66]. Chromatin accessibility and RNA abundance are often measured together to map the regulatory context of gene expression but do not include proteomic measurements [67]. Although RNA is used as an alternative marker for protein expression, additional layers of regulation occur at the chromatin level, as well as at the level of post-transcriptional and post-transcriptional control [68]. The extent to which these levels are generally regulated in health and disease remains to be fully explored. For example, whether open chromatin regions are associated with proteomics remains to be determined. Furthermore, most existing studies of *P*. *aeruginosa* infection support only one or two omics studies, and they still lack information on the molecular mechanisms underlying the multi-omics alterations in *P*. *aeruginosa* and even other bacterial infections. We still do not know which regulatory elements are involved or how chromatin status relates to other omics studies during *P*. *aeruginosa* infection. Here, we integrated epigenomic, transcriptomic, proteomic, and ubiquitin-proteomic data from mouse lung tissues and alveolar macrophages and used a systematic analytical approach to investigate the molecular mechanisms of host immunity associated with *P*. *aeruginosa* infection.

Based on the study of chromatin-accessible peak-associated DEGs in *P*. *aeruginosa*-infected lung tissues, we explored the relationship between the gene expression of key DEGs and DARs. Analysis of differential mRNA expression levels in *P*. *aeruginosa*-infected lung tissues led us to identify several genes that were significantly differentially associated with *P*. *aeruginosa* infection. In particular, including the upregulated macrophage inflammatory protein family (Cxcl3, Cxcl2, and Ccl3), changes in the expression of the macrophage inflammatory protein family may reveal an important involvement of macrophages. The overexpression of the inflammatory protein family was reported to confer an inflammatory response during infection [69]. In a genome-wide analysis of chromatin accessibility, ATAC-Seq signals showed significant enrichments in promoter-TSS and intragenic elements in *P*. *aeruginosa*-infected lung tissues. Combined with RNA-seq data, we found a positive correlation between DARs and DEGs (r = 0.41), whereby increased chromatin accessibility caused higher expression of key genes associated with *P*. *aeruginosa*-infected lung tissues (e.g., Cxcl3, Il1r2, and Acod1). Furthermore, we screened DEGs associated with changes in chromatin accessibility in *P*. *aeruginosa*-infected lung tissues for enrichment analysis and discovered that enrichment of pathways also showed inflammatory manifestations, such as NOD-like receptors and Toll-like receptors, and inflammatory pathways, such as the JAK/STAT signaling pathway. Overall, our RNA-seq results are broadly consistent with previous studies on DEGs identification and pathway enrichment analysis, and we further revealed the correlation between differential chromatin accessibility and differential genes to determine their genetic features associated with infection with *P*. *aeruginosa*. Notably, most of the identified DEGs were associated with functions revealing a phenotype of inflammation following *P*. *aeruginosa* infection of the host.

In light of the above findings and the important role of macrophages in first-line immunity, this study integrated ATAC-seq and RNA-seq data to explore the molecular changes in *P*. *aeruginosa*-infected alveolar macrophages. In particular, we found significant similarities in the molecular characteristics and functions of *P*. *aeruginosa*-infected lung tissues and mouse alveolar macrophages. To further investigate the molecular connections between tissues and cells, we specifically compared the genomic and transcriptomic molecular features of lung tissues and mouse alveolar macrophages. The results showed that ATAC-seq signals from both sources were significantly enriched in the ± 1 kb region around the TSS. Meanwhile, positive correlations in DEGs in lung tissues and alveolar macrophages were discovered, and epigenetic regulation-related alterations in ubiquitinated molecules and function were discovered utilizing DEGs gene set analysis. Subsequently, we identified multiple immune-related signaling pathways based on changes in the abundance of the proteomics and ubiquitin-proteomics of lung tissues infected with *P*. *aeruginosa*. Of these, the Positive regulation of the cytokine pathway was particularly prominent, with multi-omics changes in molecules in this pathway reflecting the inflammatory response of bacterial infection-induced host defenses. The results show that there are differences in the multi-omics expression of the 18 molecules of the Positive regulation of the cytokine pathway and that there is some consistency in the protein expression of these molecules in transcriptomics, proteomics, and ubiquitin-proteomics. Furthermore, multi-omics changes in the positive regulatory pathway of cytokines in Adam8, Anxa1, Heg1, Sptbn1, and Stat1 have been suggested to be highly correlated with inflammatory cell activation and cytokine production [47–51].

Transcription factors play an important role in the pathological progression of diseases [52]. By analyzing chromatin accessibility, we can not only identify regulatory regions but also infer TF motifs [32]. Therefore, we performed known motif analysis of DARs using HOMER to identify potential transcription factors and their target differentially expressed genes. We identified 20 DETFs whose expression changes had the same pattern in lung tissue and alveolar macrophages infected by *P*. *aeruginosa*. Using multi-omics dimensional analysis, we found that only Stat1 and Stat3 expression was remarkably altered in the four omics. Fas regulates the balance between Stat1 and Stat3 by binding and isolating Stat1 [62] and contributes to the inflammatory response by promoting T-cell differentiation through Stat3 [70]. Thus, this phenomenon may be related to the altered functional status of Fas, a downstream molecule that counter-regulates Stat1, thereby increasing the multi-omics activity of Stat3 and contributing to the development of inflammation. More importantly, this phenomenon was first identified in *P*. *aeruginosa*-infected macrophages, and the host developed an innate immune response and inflammatory response with the above molecular and functional alterations. Our results suggest that the altered multi-omics of Stat1 and Stat3 play an important role in host involvement in the immune regulation of *P*. *aeruginosa* infection and that their downstream molecules (e.g., Fas) may be involved together in the immunosuppressive and inflammatory promoting processes.

Multi-omics techniques are an emerging and effective tool for understanding and assessing the relationships between multiple molecular layers in the development of different diseases. A variety of causes and environmental changes can contribute to disease development, as well as potential molecular outcomes such as inflammatory responses and host defenses. In this study, we discovered the immunoregulatory mechanism of *P*. *aeruginosa* infection in which omics changes in central transcription factors interact with target genes to promote inflammation development at the multi-omics level. However, the present study still has some limitations. First, the relatively small sample size of some of the experiments in this study increased the experimental error to some extent. Second, our study may need deeper experimental validation to understand the underlying mechanisms of *P*. *aeruginosa* in clinical samples. Third, it is worth performing some of the key experimental validations that required the participation of male mice to exclude bias due to gender. In addition, Stat1 and Stat3 are hotspot transcription factors that act in a variety of physiological and pathological states. However, it is unclear whether their cooccurring ubiquitination modifications only affect functional alterations in *P*. *aeruginosa* infection models or also involve other pathogen infection models and even other inflammation-related diseases, and further studies are needed to clarify these unclear aspects in future studies.

In summary, by using integrated bioinformatics analysis, we provide unrecognized insights into the landscape of molecular features and functionally changed genes across multiple dimensions in *P*. *aeruginosa* infection. The important genes and regulators identified in this study provide an important resource for further uncovering the underlying mechanisms of host immune defense and are potential targets for immunodiagnosis and immunoregulatory therapy.

## Supporting information

S1 Fig Genome-wide view of chromatin accessibility in mouse lung tissue and macrophages. (A and B) Principal component analysis (PCA) and correlation analysis were performed based on the combined peak signals of all samples. (A) Principal component analysis (PCA) plot. (B) The correlation results are shown in a heatmap. (C) The distribution of ATAC-seq fragment sizes in each sample, with clear and visible signals for mono- and dinucleosomes. (D) Heatmaps indicate ATAC-seq signals across a genomic window –3 kb upstream to +3 kb downstream of the TSS.
(TIF)

S2 Fig Characterization of DEGs associated with DARs in mouse lung tissue. (A and B) Identification of infection-associated differentially expressed genes in mouse lung tissue. (A) The bars show that a total of 4229 DEGs were identified, of which 2126 were upregulated and 2103 were downregulated. (B) Heatmap showing intergroup agreement between the infected and control groups. (C) Distribution of differentially accessible regions on chromosomes in the lung tissue of mice compared to controls. (D-G) Box plots of mRNA expression levels of DEGs relative to the control (left) and infected regions (right). The notches in the boxes indicate the median. (H) Correlation analysis of downregulated DEGs with DARs. (I) DARs-associated downregulated DEGs are mainly involved in the KEGG pathway. (J) Enrichment results for key DEGs associated with DARs in the Reactome database.
(TIF)

S3 Fig Characterization of DEGs associated with DARs in mouse alveolar macrophages. (A and B) Identification of infection-associated differentially expressed genes in mouse alveolar macrophages. (A) Bar graph shows that a total of 3888 DEGs were identified, of which 2208 were upregulated and 1680 were downregulated. (B) Heatmap showing intergroup agreement between the infected and control groups. (C and D) Correlation analysis of DEGs and DARs in mouse alveolar macrophages. (C) Correlation analysis of DEGs upregulated by mouse alveolar macrophages with DARs. (D) Correlation analysis of DEGs downregulated by mouse alveolar macrophages with DARs. (E) Results of Reactome enrichment of DEGs associated with DARs in mouse alveolar macrophages. (F) Results of KEGG enrichment of DEGs associated with DARs in mouse alveolar macrophages.
(TIF)

S4 Fig Alveolar macrophage and lung tissue omics correlation GO enrichment analysis. (A) TIMER2.0 predicts macrophage infiltration abundance. (B) Gene expression values of core molecules of the ubiquitin-proteasome system are described in alveolar macrophages and lung tissues. (C) Results of proteomic downregulation of GO enrichment in mouse lung tissues. Color represents significance, and position represents the number of enriched genes. (D) Results of ubiquitinated proteomic downregulation of GO enrichment in mouse lung tissues. Color represents significance, and position represents the number of enriched genes.
(TIF)

S5 Fig The results of differential genes GO enrichment analysis in mouse alveolar macrophages and lung tissue. (A) Results of GO enrichment in mouse tissues; colors represent significance. (B) Heatmap showing inflammatory factor gene expression values in mouse tissues. (C) Results of GO enrichment in mouse alveolar macrophages; colors represent significance. (D) Heatmap showing inflammatory factor gene expression values in mouse alveolar macrophages.
(TIF)

S1 Table Annotation of differentially accessible regions (DARs) in mouse lung tissues. (CSV)

S2 Table Differentially expressed genes (DEGs) in mouse lung tissues. (CSV)

S3 Table Annotation of DARs in mouse alveolar macrophages. (CSV)

S4 Table DEGs in mouse alveolar macrophages. (CSV)

S5 Table Normalized signal values and gene expression values of genes upregulated by differentially accessible regions within mouse alveolar macrophages. (CSV)

S6 Table Normalized signal values for differentially accessible regions in mouse alveolar macrophages and tissues. (CSV)

S7 Table Differentially expressed proteins (DEPs) in mouse alveolar macrophages. (CSV)

S8 Table Differentially ubiquitinated proteins (DUPs) in mouse lung tissues. (CSV)

S9 Table DARs are enriched with known TF binding motifs in mouse lung tissue. (CSV)

S10 Table DARs are enriched with known TF binding motifs in mouse alveolar macrophages. (CSV)
