# Supplementary figures and images for: An integrated multi-omics analysis of identifies distinct molecular characteristics in pulmonary infections of Pseudomonas aeruginosa
Source: PLoS Pathog. 2023 Aug 29;19(8):e1011570. doi: 10.1371/journal.ppat.1011570 (PMC10464988; doi:10.1371/journal.ppat.1011570)

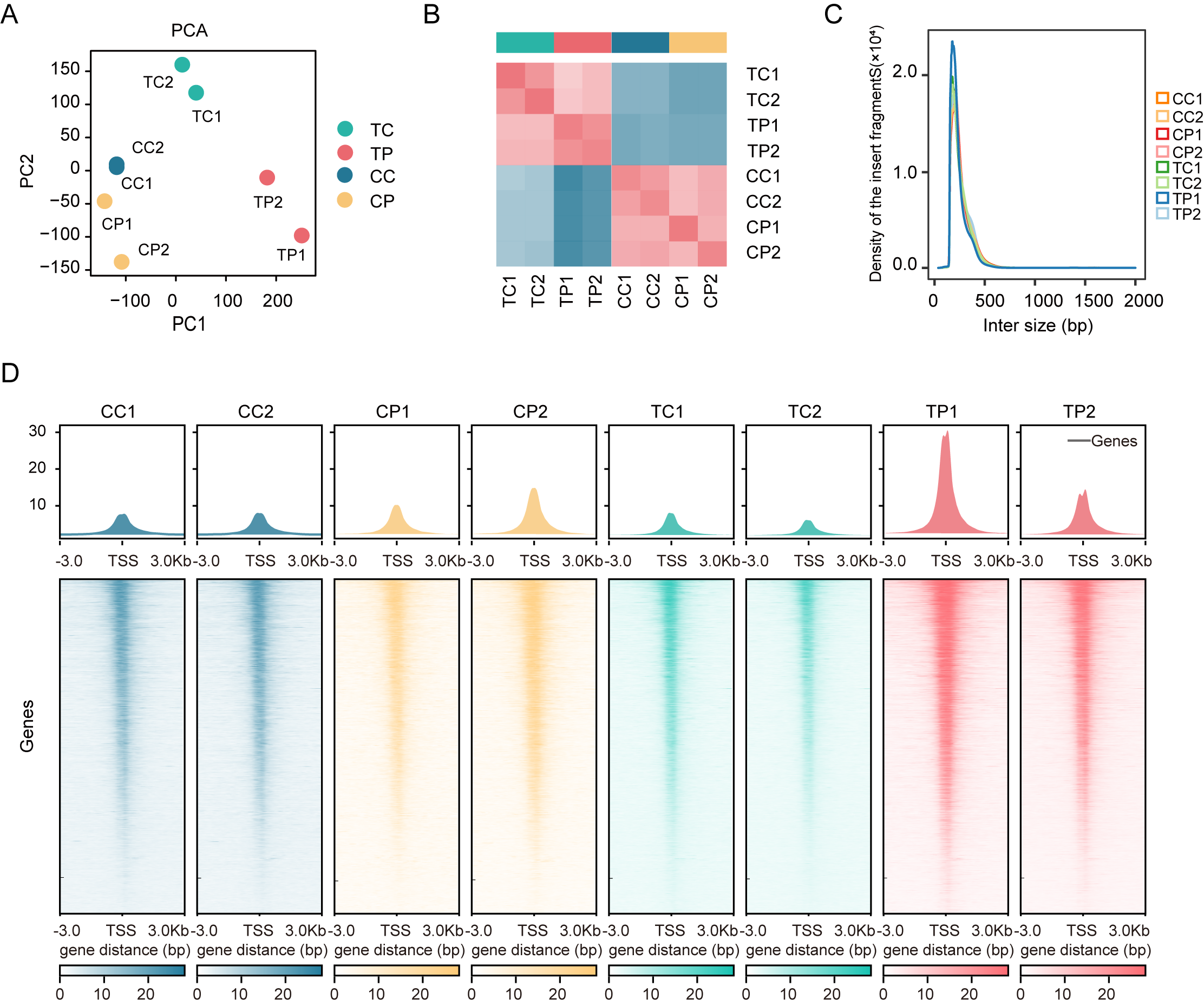

Supplement: S1 Fig — (A and B) Principal component analysis (PCA) and correlation analysis were performed based on the combined peak signals of all samples. (A) Principal component analysis (PCA) plot. (B) The correlation results are shown in a heatmap. (C) The distribution of ATAC-seq fragment sizes in each sample, with clear and visible signals for mono- and dinucleosomes. (D) Heatmaps indicate ATAC-seq signals across a genomic window –3 kb upstream to +3 kb downstream of the TSS. (TIF) [file ppat.1011570.s001.tif]

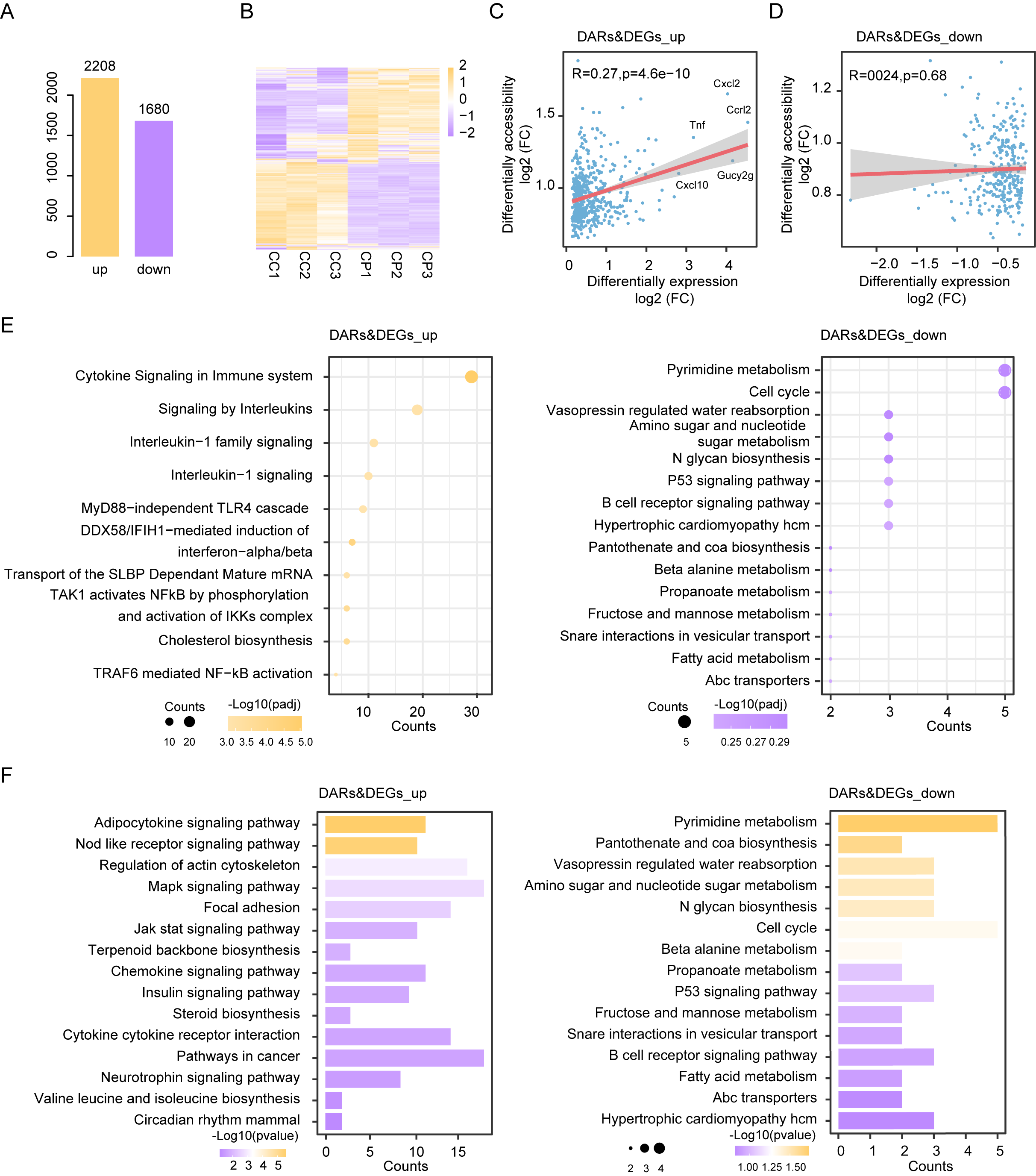

Supplement: S3 Fig — (A and B) Identification of infection-associated differentially expressed genes in mouse alveolar macrophages. (A) Bar graph shows that a total of 3888 DEGs were identified, of which 2208 were upregulated and 1680 were downregulated. (B) Heatmap showing intergroup agreement between the infected and control groups. (C and D) Correlation analysis of DEGs and DARs in mouse alveolar macrophages. (C) Correlation analysis of DEGs upregulated by mouse alveolar macrophages with DARs. (D) Correlation analysis of DEGs downregulated by mouse alveolar macrophages with DARs. (E) Results of Reactome enrichment of DEGs associated with DARs in mouse alveolar macrophages. (F) Results of KEGG enrichment of DEGs associated with DARs in mouse alveolar macrophages. (TIF) [file ppat.1011570.s003.tif]

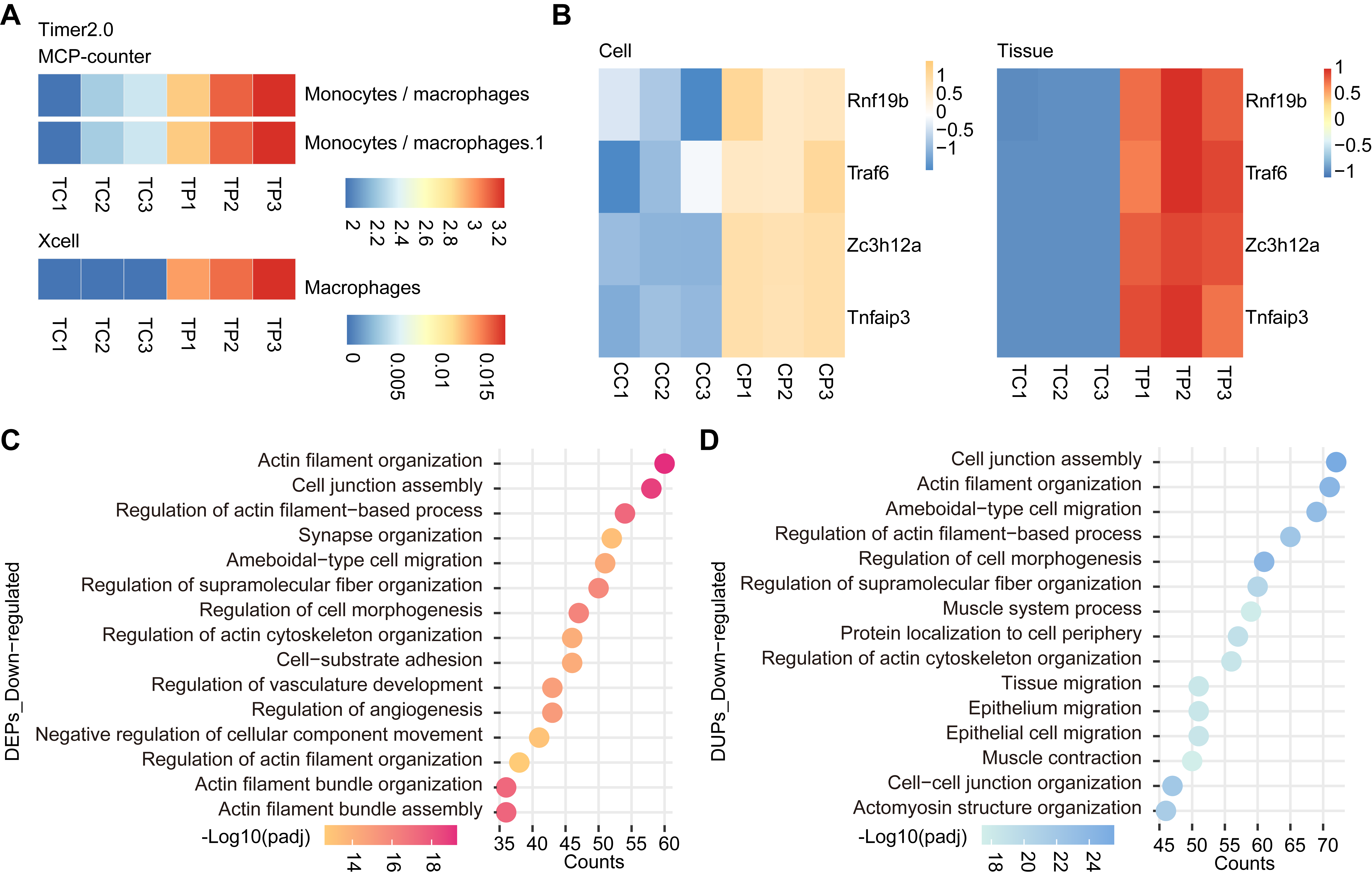

Supplement: S4 Fig — (A) TIMER2.0 predicts macrophage infiltration abundance. (B) Gene expression values of core molecules of the ubiquitin-proteasome system are described in alveolar macrophages and lung tissues. (C) Results of proteomic downregulation of GO enrichment in mouse lung tissues. Color represents significance, and position represents the number of enriched genes. (D) Results of ubiquitinated proteomic downregulation of GO enrichment in mouse lung tissues. Color represents significance, and position represents the number of enriched genes. (TIF) [file ppat.1011570.s004.tif]

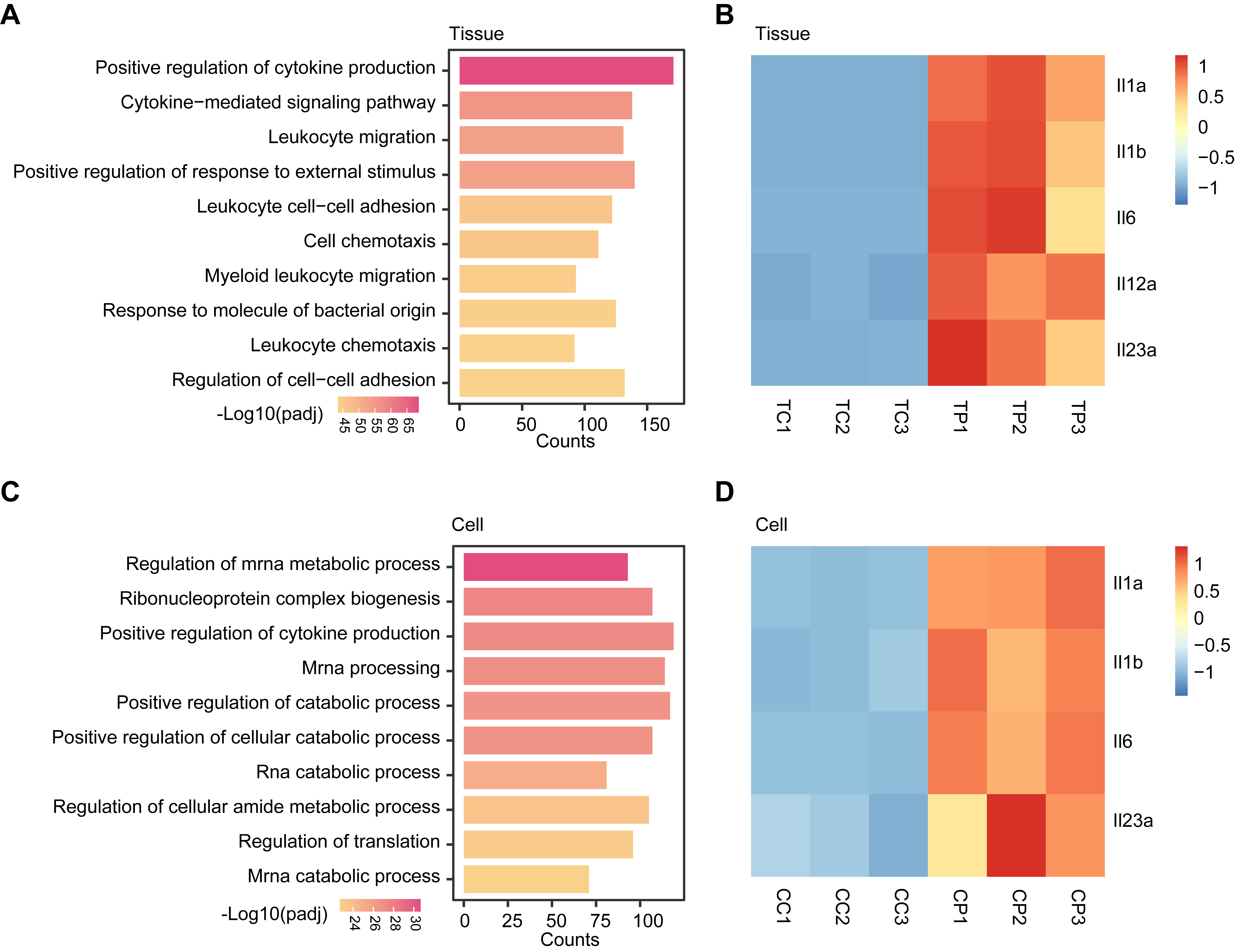

Supplement: S5 Fig — (A) Results of GO enrichment in mouse tissues; colors represent significance. (B) Heatmap showing inflammatory factor gene expression values in mouse tissues. (C) Results of GO enrichment in mouse alveolar macrophages; colors represent significance. (D) Heatmap showing inflammatory factor gene expression values in mouse alveolar macrophages. (TIF) [file ppat.1011570.s005.tif]
